# Frequency of Causes of Portal Hypertension in Children

**DOI:** 10.7759/cureus.25934

**Published:** 2022-06-14

**Authors:** Kumar Sooraj, FNU Shivani, Mahnoor Hassan Khan, Rahul Robaish Kumar, Shilpa Bai, Helai Hussaini, FNU Rakesh, Amna Jamil, Hareem Arshad, Sidra Naz

**Affiliations:** 1 Internal Medicine, Jinnah Sindh Medical University, Karachi, PAK; 2 Internal Medicine, Peoples University of Medical and Health Sciences, Nawabshah, PAK; 3 Internal Medicine, Abbasi Shaheed Hospital, Karachi, PAK; 4 Internal Medicine, Jinnah Postgraduate Medical Centre, Karachi, PAK

**Keywords:** medicine-pediatrics, liver cirrhosis, biliary atresia, portal venous thrombosis, portal hypertension

## Abstract

Introduction

The most common etiology of portal hypertension (PH) in children is obstruction at the presinusoidal or sinusoidal level. In addition, portal vein thrombosis (PVT) and biliary atresia are the most prevalent extrahepatic causes. This study aims to evaluate all the possible etiologies leading to PH in the pediatric population and provide the most common cause associated with this condition along with the age group most frequently affected by it.

Material and Methods

From January 2018 to December 2020, a cross-sectional study was carried out in tertiary care hospitals in Pakistan. A total of 100 children, both male and female, aged one month to 15 years and diagnosed with PH, were enrolled for the evaluation for the causes of PH. The Statistical Package for the Social Sciences (SPSS), version 20, was used to analyze the data.

Results

The mean age of enrolled participants was 9.01 ± 2.81 years. It was found that PVT (63%) was the most common cause of PH, followed by liver cirrhosis (19%) and biliary atresia (18%). Age of more than eight years was significantly associated with PVT (*p*-value: 0.007).

Conclusion

In children, PH may be caused by a wide range of etiologies. It is imperative to understand the underlying etiologies contributing to PH for proper guidance and management, prevention, and overall outcomes.

## Introduction

The term "portal hypertension" (PH) refers to the presence of portal resistance or a rise in blood flow in the portal venous system, that is, both low baseline portal venous pressure ranging from 7 to 10 mmHg and hepatic venous pressure gradient (HVPG), which is the difference between the wedged hepatic venous pressure and the free hepatic venous pressure, ranging from 1 to 4 mmHg. PH is defined as a portal pressure greater than 10 mmHg or HVPG greater than 4 mmHg [1,2]. Although it is estimated that portal pressure above 12 mmHg or higher is associated with complications but it is rarely measured directly and is rather seen through events of pathological changes such as splenomegaly, development of varices, esophageal ascites, hepatopulmonary syndrome, and cirrhosis [3].

PH can occur due to intrahepatic or extrahepatic disruption of portal blood flow [4]. The cause of PH has been classified based on sites of increased resistance, including prehepatic, hepatic, or posthepatic, which is further classified into presinusoidal, sinusoidal, and postsinusoidal as well as the presence of physiological alterations like increased mesenteric blood flow, increased hepatic venous wedge pressure, and so on [5,6]. In children, the most common etiology of PH is obstruction at the presinusoidal or sinusoidal level, which includes diseases such as cirrhosis, congenital hepatic fibrosis, nodular regenerative hyperplasia, and possibly cystic fibrosis [4]. In addition, portal vein thrombosis (PVT) and biliary atresia are the most prevalent extrahepatic causes [5]. Previous portal phlebitis or previous localized pathology or surgery, or systemic factors, such as dehydration or a prothrombotic state, can be the possible reasons behind thrombosis, but usually, no cause has been identified and it has remained idiopathic in the majority of the cases [6]. Clinical presentation of PH in children also depends on the underlying etiology, for example, in cirrhosis, upper gastrointestinal (GI) bleeding is most commonly seen, while jaundice and ascites are frequently observed in PVT [7].

To the best of our knowledge, no local data is available related to the causes of PH in children. The purpose of our study is to evaluate all the possible etiologies leading to PH in our local pediatric population and provide the most common cause associated with this condition along with the age group most frequently affected by it.

## Materials and methods

From January 2018 to December 2020, a cross-sectional study was carried out in tertiary care pediatric hospitals in Pakistan. A total of 100 children, both male and female, aged one month to 15 years and diagnosed with PH, were enrolled in the pediatric outpatient department using a nonprobability, consecutive sampling technique. Ethical approval for the study was obtained from the institutional review board of the People's University of Medical and Health Sciences (PUMHSW/IRB/IM/165). Participants with congenital anomalies, such as congenital heart disease, were not included in the study. A sonologist diagnosed PH as portal pressure greater than 10 mmHg using Doppler ultrasound.

The process and protocol were described to the participants' parents, and their informed consent was obtained. Participants over the age of five had their consent recorded along with their parents' consent to participate in the study. Following registration, patients underwent a thorough medical history and physical examination to check for signs of PH like ascites, splenomegaly, jaundice, and bleeding. Patient samples were taken for complete blood counts and liver function tests once they were registered in the research. Ultrasound abdomen and color Doppler ultrasound were taken to determine portal vein pressure to classify PVT, liver cirrhosis, and biliary atresia as causes of PH (Table 1).

Table 1: Definition of causes of PH

**Table 1:** Definition of causes of PH PH, portal hypertension; PVT, portal vein thrombosis; ALT, alanine transaminase.

Term	Definition
PVT	A clot of blood obstructing the flow of blood in the portal vein as evident on color Doppler ultrasound
Liver cirrhosis	Labeled as the presence of all of the following: coarse parenchymal echogenicity and irregular margins of liver on ultrasound abdomen; serum albumin <3.5 g/dl; serum ALT >40 IU/L
Biliary atresia	Congenitally scared or blocked major bile duct with triangular cord sign on ultrasound

Data analysis was done using the Statistical Package for the Social Sciences (SPSS, New York, USA), version 20. Mean and standard deviation were calculated for age, portal pressure, and course of illness. For categorical variables, such as age groups, gender, residential status, maternal literacy level, family income, and causes of PH, frequencies and percentages were determined. Stratification was used to regulate effect modifiers such as age, gender, and length of sickness. After stratification, a chi-square test was used to evaluate how they affected the outcome, that is, causes of PH. A *p*-value <0.05 was considered statistically significant and the null hypothesis is void.

## Results

The mean age of enrolled participants was 9.01 ± 2.81 years. Enrolled participants included 63% of males and 69% belonged to the age group of more than 8 years. The demographics are discussed in Table 2.

**Table 2:** Characteristics of participants

Characteristics	Value (*n* = 100)
Mean age (years)	9.01 ± 2.81
Age group	
Below 8 years (%)	31 (31%)
More than 8 years (%)	69 (69%)
Gender	
Male (%)	63 (63%)
Female (%)	37 (37%)

PVT (63%) was found to be the most common cause of PH, followed by liver cirrhosis (19%) (Table 3).

**Table 3:** Causes of PH PH, portal hypertension; PVT, portal vein thrombosis.

Causes of PH	N (%)
PVT	63 (63%)
Liver cirrhosis	19 (19%)
Biliary atresia	18 (18%)

Causes of PH were more prevalent in male participants; however, the association was not significant. PVT was found to be significantly associated in participants with more than eight years of age (p-value: 0.007). Stratification of causes of PH based on gender and age is presented in Table 4.

**Table 4:** Stratification of causes based on gender and age PH, portal hypertension; PVT, portal vein thrombosis.

Causes of PH, *n* (%)	Gender (*n*)			Age of participant (*n*)		
	Male (63)	Female (37)	*p*-Value	Less than 8 years (24)	More than 8 years (76)	*p*-Value
PVT 63 (63%)	39 (61.92%)	24 (64.86%)	0.08	12 (50%)	51 (67.11%)	0.007
Liver cirrhosis 19 (19%)	12 (19.04%)	07 (18.92%)	0.98	06 (25%)	13 (17.11%)	0.95
Biliary atresia 18 (18%)	12 (19.04%)	06 (16.22%)	0.72	06 (25%)	12 (15.78%)	0.81

## Discussion

Our study demonstrated that PH was more prevalent in males, and participants above eight years of age with PVT being the most common cause. The frequency of causes differs between children and adults as well as the physiological variations between both populations. Several studies have endorsed the pattern of PH to differ between adults and children, that is, intrahepatic in adults and extrahepatic in children [8-11]. In children, the most common pathophysiology of PH relates to partial or complete blockage of the portal trunk [12].

In Egyptian children, similar findings were seen during a cross-sectional study. PVT was found to be the most common cause of PH, and congenital hepatic fibrosis was the second most common cause [13]. A study carried out in India among children <14 years of age also showed extrahepatic portal venous obstruction as the most common cause of PH, followed by cirrhosis [14]. However, another study conducted in India reported noncirrhotic portal fibrosis as the commonest cause of PH in children aged between 14 months and 10 years [15]. According to an Iranian study conducted among children, 93.3% of PH cases were due to intrahepatic causes, cryptogenic cirrhosis was the most common cause followed by biliary atresia and Wilson’s disease, and only 4.4% were attributable to PVT [16]. Hence, by the literature available, it can be observed that the etiology of PH in children varies in prevalence due to differences in genetic, environmental, regional, and ethnic factors.

The most common risk factor for developing PVT in childhood is umbilical vein catheterization [17]. Other common causes include neonatal sepsis, abdominal infection, cardiovascular malformation, coagulation disorders, and abdominal surgery, as opposed to adults where the main factor for PVT is hypercoagulability [17]. Cirrhosis, another leading etiology of PH, is caused by biliary atresia and genetic metabolic diseases in infancy, whereas in older children, it is mostly caused by autoimmune hepatitis, Wilson’s disease, alpha-1-antitrypsin deficiency, and primary sclerosing cholangitis [18].

Two common presentations of PH in children are splenomegaly and bleeding, secondary to varices. Other features include ascites, jaundice, thrombocytopenia, and leukopenia. Such presentation should prompt investigation with ultrasound abdomen, color Doppler ultrasound, and endoscopy to establish the diagnosis [19]. Patients undergoing umbilical vein catheterization should be monitored for the development of PVT.

To the best of our knowledge, this is the first study from this region to study the etiology of PH in children. However, since our data collection was limited to hospitals from one region only, care should be taken while inferring the results to the general population. Another limitation to our study was that equal gender proportions of the sample were not taken, and hence, commenting on the increased prevalence of PVT in males when compared to females might show bias. Furthermore, it is a cross-sectional study, and hence, the cause-and-effect relationship could not be established.

## Conclusions

In children, PH may be caused by a wide range of etiologies. In our pediatric population, PVT was found as the most common cause of PH, followed by liver cirrhosis and biliary atresia. An increase in the incidence of PVT was seen in patients older than 8 years of age. Following the detection of PH, the monitoring and timely management of preventable problems contributing to PH should be implemented. Management can range from routine screenings and medications to endoscopic and surgical options to ensure better prognosis and long-term outcomes. The scientific rationale of this study is to help identify the common precipitants of PH in children to direct screening and investigative efforts. Such patients should be monitored and screened for the development of PVT to prevent further complications. More research is needed to better understand the long-term effects of this demographic as they enter adulthood. A large-scale study could also help researchers better determine mortality predictors and intervention criteria, as well as get a better knowledge of how the disease progresses.
